# Implantable Cardioverter-Defibrillator in Dilated Cardiomyopathy after the DANISH-Trial Lesson. A Poly-Parametric Risk Evaluation Is Needed to Improve the Selection of Patients

**DOI:** 10.3389/fphys.2017.00873

**Published:** 2017-10-31

**Authors:** Marcello Disertori, Michela Masè, Marta Rigoni, Giandomenico Nollo, Eloisa Arbustini, Flavia Ravelli

**Affiliations:** ^1^Healthcare Research and Innovation Program, Autonomous Province of Trento and Bruno Kessler Foundation, Trento, Italy; ^2^Department of Cardiology, Santa Chiara Hospital, Trento, Italy; ^3^Laboratory of Biophysics and Biosignals, Department of Physics, University of Trento, Trento, Italy; ^4^Centre for Inherited Cardiovascular Diseases, University Hospital Policlinico San Matteo, Pavia, Italy

**Keywords:** ventricular arrhythmias, sudden cardiac death, implantable cardioverter-defibrillator, dilated cardiomyopathy, genetics, heart failure, late gadolinium enhancement

## Background

The implantable cardioverter-defibrillator (ICD) is widely utilized in clinical practice, and its efficacy in reducing sudden cardiac death (SCD) has been proven by a number of studies (Moss et al., 2002; Bardy et al., 2005). The current guidelines of the European Society of Cardiology (ESC) recommend (class I, level of evidence B) ICD for primary prevention of SCD in patients with non-ischemic dilated cardiomyopathy (DCM), symptomatic heart failure (HF) with New York Heart Association class II-III, and an ejection faction (EF) ≤35% (Priori et al., 2015). Although the EF value is still considered the major determinant for ICD implantation, it displays low sensitivity and specificity as a risk marker for SCD (Disertori et al., 2016a). The recent publication of the Danish Study to Assess the Efficacy of ICDs in Patients with Non-ischemic Systolic Heart Failure on Mortality (DANISH) trial (Køber et al., 2016), which showed the absence of a reduction of total mortality in the patients randomized to ICD, is forcing us to rethink the selection criteria for ICD therapy in DCM patients.

## EF as a risk marker: conflicting evidence from randomized trials

The guidelines recommendations in DCM patients are based on the results of two randomized controlled trials (RCTs) [the Defibrillators in Non-Ischemic Cardiomyopathy Treatment Evaluation (DEFINITE) (Kadish et al., 2004) and the SCD in Heart Failure Trial (SCD-HeFT) (Bardy et al., 2005)], which were available at the time of publication of the guidelines. The trials showed a trend toward a reduction of mortality from any cause in the ICD arm, which gained significance when the two studies were meta-analyzed (Theuns et al., 2010). Of note, both studies were performed in the 2,000 s, and the patients were enrolled between 1997 and 2002. The recent DANISH trial enrolled patients in Denmark between 2008 and 2014 (Køber et al., 2016). All patients were treated according to the modern HF protocols. The rationale of the trial was “the limited evidence of a benefit from the implantation of an ICD in patients with chronic non-ischemic HF,” which ethically supported the randomization to ICD or no ICD of 1,116 patients with EF ≤ 35%, regardless of the ICD indication of the available guidelines (Zipes et al., 2006). In both ICD and control arms, 58% of the patients received cardiac resynchronization therapy (CRT). In a median follow-up period of 5.6 years, mortality from any cause was similar in the ICD and control groups (hazard ratio 0.87; 95% confidence interval: 0.68–1.12; *p* = 0.28). As well, subgroup analysis did not show any statistically significant difference in total mortality between the patients with CRT-defibrillator and CRT-pacemaker (*p* = 0.59), leaving unclear whether patients eligible for CRT should routinely receive an ICD.

The neutral result of the DANISH trial reinforces the doubts about the benefit of ICD therapy in patients with DCM, selected on the basis of the EF value. Although the DANISH trial was conducted on the homogeneous Danish population, it is likely that the results are extendable to populations outside Denmark. Indeed, the neutral result of the trial is mainly related to a statistically significant reduction of SCD risk due to the modern therapeutic approach to HF (McMurray, 2016). The SCD risk reduction by modern HF therapy is a general result, as testified by a recent extensive clinical study (Shen et al., 2017), which comprised data from 40,195 HF patients with depressed EF, enrolled in 12 clinical trials from 1995 to 2014 in a large geographic area including Europe and North America. The remarkable evolution of HF treatment and the consequent significant decrease in the risk of both cardiac death (Rush et al., 2015) and SCD (Shen et al., 2017) should be taken into account also when interpreting results from recent meta-analyses, combining the DANISH trial with previous RCTs. Although these meta-analyses demonstrated a statistically significant reduction of total mortality in patients undergoing ICD implantation (Al-Khatib et al., 2017; Golwala et al., 2017; Shun-Shin et al., 2017), their results may be biased by the mixed population analyzed, since the meta-analyzed studies were carried out up to 12 years apart and included series in which HF treatment evolved and improved.

The results of these meta-analyses are not sufficient to reduce the impact of the DANISH trial, as testified by a recent survey of the European Heart Rhythm Association (Haugaa et al., 2017), which analyzed the changes in ICD indications in patients with DCM in the post-DANISH era. Among the 48 European Centers from 17 different countries, which answered the queries, 46% declared to have changed their ICD indications after the DANISH trial publication, and 33% reported the need of further evidence. The survey results make manifest the urgent need, perceived by clinical cardiologists, of a better ICD patient selection. According to the DANISH results, in patients with non-ischemic HF and EF ≤ 35%, treated with current therapy, the occurrence of all-cause mortality and SCD were 5.0 and 1.8 events per 100 patient-years in the control group vs. 4.4 and 0.9 events in the ICD arm. Considering the relatively low risk of SCD, it is unlikely that ICD therapy, which acts solely on SCD, can significantly affect all-cause mortality in these patients. Indeed, the number needed to treat to prevent one death in a follow-up of 5.6 years was very high (56 patients).

The problem today is not to evaluate the effectiveness of ICD therapy, but rather to identify the patients who can best benefit from ICD primary prevention. Indeed, the majority of patients who received an ICD according to the current guidelines, do not experience appropriate ICD interventions, thus having no benefit from the device (Weeke et al., 2013; Sabbag et al., 2015) while being exposed to ICD-related adverse events (van der Heijden et al., 2015). In addition, several SCDs occur in patients with moderately depressed EF, who are not included in the current indications for ICD therapy (Wellens et al., 2014). The ESC proposal “for a revised definition of DCM and hypokinetic non-dilated cardiomyopathy” (Pinto et al., 2016) arose precisely from the evidence that there are DCM patients in whom left ventricular dysfunction and dilatation are still mild, but the absence of arrhythmogenic risk is not guaranteed. Finally, the cohorts of patients with EF ≤ 35% are inevitably exposed to the high competitive risk of death due to the evolution of HF or to non-cardiac causes (65% of total mortality in the control group of the DANISH trial). With the current recommendations, the risk is wasting money and harm patients, implanting ICDs in patients who will not benefit from them, and withholding ICDs from patients whose survival could be improved by the treatment. In the DANISH trial, 5.9% of the patients received inappropriate shocks, which may lead to quality of life impairment and potentially increase mortality (Poole et al., 2008; Tung et al., 2008; Sweeney, 2012).

As recently suggested, a poly-parametric evaluation may help to improve the appropriateness of ICD therapy by selectively identifying those patients who may have the highest benefit from it (Disertori et al., 2013, 2016a). Several invasive and non-invasive markers of arrhythmic risk have been proposed (Disertori et al., 2016a). Among these, fibrosis identification by late gadolinium enhancement cardiac magnetic resonance (LGE-CMR) seems the most promising marker in DCM patients (Disertori et al., 2017).

## LGE assessment of ventricular fibrosis

LGE-CMR is a feasible test to assess the presence of fibrosis. Ventricular fibrosis promotes ventricular arrhythmias by harboring critical reentrant pathways and favoring the emergence of arrhythmogenic triggers (Morita et al., 2014; Nguyen et al., 2014). Differently from ischemic cardiomyopathy in which ventricular fibrosis is present in almost all the patients, the incidence of fibrosis in DCM is ~30–40% (Disertori et al., 2016b). Large prospective observational studies with adequate follow-up (Gulati et al., 2013; Disertori et al., 2017; Halliday et al., 2017) and extensive meta-analyses (Disertori et al., 2016b; Di Marco et al., 2017) have shown that the absence of fibrosis in DCM patients predicts a relatively low risk of SCD, while the presence of fibrosis predicts a relatively high risk of SCD, irrespective of the EF value. In a recent meta-analysis of 2,948 patients with DCM (Di Marco et al., 2017), the arrhythmic endpoint (SCD, ventricular tachyarrhythmias, appropriate ICD therapy) occurred in 350 patients. The annualized event rate was 6.9% in patients with ventricular fibrosis vs. 1.6% in patients without fibrosis, with no significant difference in the subgroups of studies with mean EF < 35% vs. EF > 35%. Given the small annualized arrhythmic event rate (1–2%) in patients with EF < 35% and absence of fibrosis, a significant ICD benefit is unlikely despite the EF value. On the other end, patients with EF between 36 and 50% and ventricular fibrosis had a relatively high annualized arrhythmic event rate (7.3%), and may be potential candidates for ICD therapy.

Despite the large amount of observational studies on the predictive power of myocardial fibrosis for ventricular tachyarrhythmias in DCM patients, no RCTs have been published yet. Only the Cardiovascular Magnetic Resonance-GUIDEd (CMR-GUIDE) trial (Selvanayagam et al., 2017) is currently randomizing ischemic and non-ischemic cardiomyopathy patients with moderate systolic dysfunction (EF 36–50%) and presence of fibrosis at LGE, to either ICD or implantable loop recorder. However, the trial's estimated completion date is December 2020. The lack of RCT results on LGE should not restrain the implementation of LGE assessment into risk stratification criteria for ICD therapy in DCM patients. In the hierarchy of primary research designs RCTs are considered the highest grade of evidence, whereas observational studies are regarded of lower validity. Nevertheless, systematic reviews assessing the impact of study design on the estimated effect measures and the risk of adverse effects, concluded that there was no significant difference between the estimates provided by RCTs vs. observational studies (Concato et al., 2000; Anglemyer et al., 2014). According to this growing evidence, prospective registries and meta-analyses of well-conducted observational studies may provide a valuable alternative to measure the effectiveness of an intervention in “real world” scenarios, and an adequate source of evidence for decision-making, as highlighted also in the ESC guidelines on ventricular arrhythmias (Priori et al., 2015). Recent observational studies and meta-analyses supporting the prognostic value of LGE as decision criterion for ICD therapy in DCM patients should thus be considered evidence for rethinking ICD indications.

## Clinical and molecular genetic profiling

**Figure 1:**
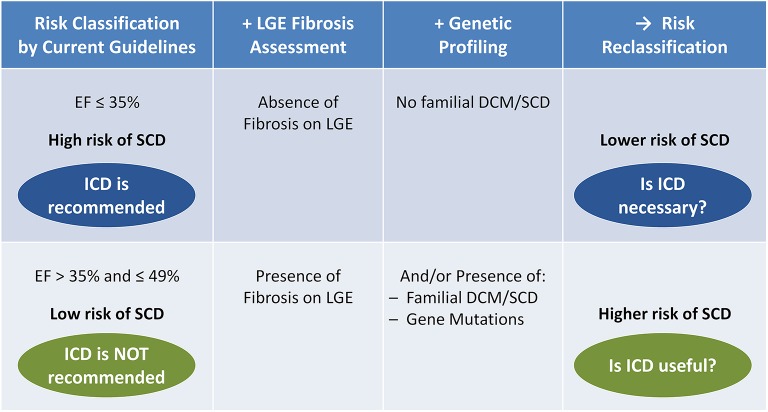
Potential reclassification of the risk of sudden cardiac death (SCD) by late gadolinium enhancement (LGE) fibrosis assessment combined with clinical and molecular genetic profiling, in patients with dilated cardiomyopathy (DCM) and heart failure (NYHA class II-III), under optimal medical therapy for at least 3 months, and with life expectancy >1 year. Among patients with severely depressed left ventricular function [ejection fraction (EF) ≤ 35%], a negative LGE test (absence of fibrosis) combined with the absence of familial DCM/SCD may identify a subgroup of patients at lower risk of SCD, in which implantable cardioverter-defibrillator (ICD) may be not necessary (blue). Among patients with moderately depressed left ventricular function (EF > 35% and ≤ 49%), a positive LGE test (presence of fibrosis) and/or the presence of familial DCM/SCD or pathological gene mutations may identify a subgroup at higher risk of SCD, in which ICD may be useful (green).

The clinical diagnosis of DCM is currently based on its morpho-functional phenotype, but not on etiopathogenetic bases. Beyond the distinction between non-genetic and genetic causes, a recent proposal by the ESC Myocardial and Pericardial Diseases Working Group has highlighted the distinction between the phenotypes of DCM and hypokinetic non-dilated cardiomyopathy (Pinto et al., 2016). DCM is genetic in a large proportion of cases and is an example of genetic heterogeneity, with many disease, and candidate genes involved (Arbustini et al., 2014). As suggested by the current guidelines (Priori et al., 2015), both familial history and genotyping may aid in the diagnostic and prognostic classification of patients with familial DCM, particularly for the identification of a family history of SCD and of pathological mutations (e.g., Lamin A/C; van Rijsingen et al., 2012). Both these factors could help identifying patients at higher risk of SCD even in the presence of a moderately depressed EF. Figure 1 shows a proposal of expansion of the criteria for SCD primary prevention, which combines left ventricular EF with ventricular fibrosis assessment by LGE-CMR and family history of DCM and/or SCD (Disertori et al., 2016a; Arbustini et al., 2017).

## Conclusions

In conclusion, ICD therapy works, but it is challenging to find out who may benefit from it. Accumulating evidence by meta-analyses of well-performed observational studies pointed out the predictive power of LGE-CMR for arrhythmic events in DCM patients. We believe that the time is ripe for a poly-parametric approach to risk stratification, which should include the assessment of ventricular fibrosis by LGE, in addition to EF and the genetic profile of the patient when available. This could significantly improve the appropriateness of ICD therapy in DCM patients, going in the direction of personalized and precision medicine that is, in fact, the epoch of today's medicine.

## Author contributions

MD and EA conceived and designed the study. MM and MR extracted and analyzed the data. MD, FR, GN, and EA interpreted the data. MD, MM, and EA drafted the manuscript. MR, FR, and GN critically revised the manuscript for significant intellectual content. All authors read and approved the final version of the manuscript.

### Conflict of interest statement

The authors declare that the research was conducted in the absence of any commercial or financial relationships that could be construed as a potential conflict of interest.
